# Pricing and reimbursement mechanisms for advanced therapy medicinal products in 20 countries

**DOI:** 10.3389/fphar.2023.1199500

**Published:** 2023-11-28

**Authors:** Juan Carlos Rejon-Parrilla, Jaime Espin, Sarah Garner, Stanislav Kniazkov, David Epstein

**Affiliations:** ^1^ Health Technology Assessment Area (AETSA), Andalusian Public Foundation Progress and Health (FPS), Seville, Spain; ^2^ Andalusian School of Public Health, Granada, Spain; ^3^ Instituto de Investigación Biosanitaria ibs, Granada, Spain; ^4^ CIBER of Epidemiology and Public Health (CIBERESP), Madrid, Spain; ^5^ Cátedra de Economía de la Salud y Dirección de Organizaciones Sanitarias (Esalud2), Granada, Spain; ^6^ World Health Organization Regional Office for Europe, Copenhagen, Denmark; ^7^ Department of Applied Economics, University of Granada, Granada, Spain

**Keywords:** advanced therapy medicinal products (ATMPs), pricing and reimbursement (P&R), pharmaceutical policy, survey, health technology assessment (HTA)

## Abstract

**Introduction:** Advanced Therapy Medicinal Products are a type of therapies that, in some cases, hold great potential for patients without an effective current therapeutic approach but they also present multiple challenges to payers. While there are many theoretical papers on pricing and reimbursement (P&R) options, original empirical research is very scarce. This paper aims to provide a comprehensive international review of regulatory and P&R decisions taken for all ATMPs with centralized European marketing authorization in March 2022.

**Methods:** A survey was distributed in July 2022 to representatives of 46 countries.

**Results:** Responses were received from 20 countries out of 46 (43.5%). 14 countries reimbursed at least one ATMP. Six countries in this survey reimbursed no ATMPs.

**Conclusion:** Access to ATMPs is uneven across the countries included in this study. This arises from regulatory differences, commercial decisions by marketing authorization holders, and the divergent assessment processes and criteria applied by payers. Moving towards greater equality of access will require cooperation between countries and stakeholders, for example, through the WHO Regional Office for Europe’s Access to Novel Medicines Platform.

## 1 Introduction

Advanced Therapy Medicinal Products (ATMPs) are medicines for human use that are based on genes, tissues or cells (EMA, 2022a). Some of these therapies hold great potential for patients without an effective current therapeutic approach (Hanna et al., 2016; Lamas-Díaz and Hernández-García, 2020). Development is rapid in this area. By October 2022, 19 ATMPs had received full, conditional or exceptional marketing authorization (MA) in the European Union (EU) (Aguilera-Cobos et al., 2022). The Food and Drugs Administration (FDA) forecasts that by 2025 they will approve every year between 10 and 20 cell and gene therapies (Food and Administration, 2019). However, the individual companies choose whether to submit products for regulation, to the FDA or to other regulatory bodies in other regions, as well as for registration and reimbursement in particular countries. For example, whilst a product may have a central marketing authorization, the companies can then decide when and where to launch or file for reimbursement.

The generation of evidence in therapeutic areas where there is an unmet medical need can be challenging (Vreman et al., 2019). The PRIority MEdicines (PRIME) scheme was developed by the European Medicines Agency (EMA) to enhance technical support for the development of medicines that target an unmet medical need. Many ATMPs target unmet needs. Almost half (45%) of PRIME designations (Aguilera-Cobos et al., 2022)–combining medicines that were once granted PRIME designation but that are no longer in the scheme and therapies that are in the scheme at the time of writing–were ATMPs (EMA, 2022c) (Supplementary Annex S1). Furthermore, ATMPs, up to now, have almost all been designated as orphan drugs for rare diseases (14 out of the 19 approved by the EMA).

In order to facilitate early access for patients, where a product addresses an unmet need, regulators can give a conditional MA on the basis of early data, providing certain conditions are met including the provision of further evidence (Bloem et al., 2023). However, this often means that MA holders then file for reimbursement with insufficient evidence to support the claim of cost-effectiveness (Bloem et al., 2023), particularly in the long-term. As these medicines are often priced highly this creates high financial and clinical uncertainty and risk for payers. Outcomes-based (or pay-for-performance (P4P)) arrangements offer instruments that can mitigate financial risk, limit the patient population and generate further evidence. Qualitative research suggests that some experts view P4P schemes as potential enablers for MA holders to meet many of their strategic goals (Wenzl and Chapman, 2019). Early access allows sales to be initiated sooner in the product life cycle, allowing earlier returns on capital.

Whilst regulatory policies are being adopted in Europe to facilitate the accelerated approval of ATMPs (Fürst-Ladani et al., 2023), the complexities of the existing pathways are often seen as a barrier by therapy developers (Pizevska et al., 2022). However, if marketing authorization is successfully obtained, gaining access to a market where there was previously unmet need can set up the product as the market leader, develop economies of scale, and potentially establish it as the new standard of care (“first-mover advantage”). Furthermore, sales can be made without changing the “official” price of the product in that country (i.e., the net price of a therapy in a country does not need to be the same as its list price (Dubois, 2019)), which is advantageous for the MA holder in countries that adopt external reference pricing. Whilst that can be attractive to manufacturers, it can raise questions about equity in access (Kanavos et al., 2020).

The way ATMPs are administered has relevance for decision making both from clinical and reimbursement perspectives. Unlike most medicines, which can be withdrawn if no response is achieved, gene therapies are one-off treatments. Out of the 15 indications (13 ATMPs) in our sample, 14 are intended for single administration (Supplementary Annex S1). Due to the early and often sparse evidence base at launch, the clinical and economic data that reaches Health Technology Assessment (HTA) and reimbursement stage can be insufficient for healthcare systems to assess their added therapeutic value with certainty (Angelis et al., 2020; Lloyd-Williams and Hughes, 2021) and to negotiate value-based prices (Hanna et al., 2018). The difficulty of demonstrating value to payers, very small fragmented markets, and manufacturing and logistical difficulties have been cited as reasons for the withdrawal of some ATMPs from the market in Europe (Aguilera-Cobos et al., 2022).

Payers handling the difficult task of managing financial risk and uncertain evidence, where it exists, need to embed risk management strategies into their pricing and reimbursement (P&R) decision making processes, and they often do so through special pricing mechanisms (Hanna et al., 2018; Gonçalves, 2021; Jørgensen and Kefalas, 2021). While there are many theoretical papers on P&R options (Carr and Bradshaw, 2016; Godman et al., 2018; Gonçalves, 2021; Ádám et al., 2022), original empirical research is very scarce. The Organization for Economic Co-operation and Development (OECD) conducted a survey of experts on the use of managed entry agreements (MEA) in 12 countries (Wenzl and Chapman, 2019) but did not deal with specific therapies. A few papers describe country experiences of P&R arrangements (Jørgensen et al., 2020; Facey et al., 2021; Jørgensen and Kefalas, 2021; Ronco et al., 2021). This paper aims to provide a comprehensive international review of regulatory and P&R decisions taken for all ATMPs with European marketing approval in March 2022. We consider regulatory approval, reimbursement status, use of special P&R arrangements (type and aims) and arrangements for further evidence collection and re-assessments.

## 2 Methods

A survey was distributed in July 2022 to 46 countries (see Supplementary Annex S2) through the Pharmaceutical Pricing and Reimbursement Information (PPRI) Network, a unique collaboration of pharmaceutical P&R authorities with 50 members from national competent bodies (mostly European) and international institutions. The PPRI enables members to exchange information and data on P&R decisions and policies (Vogler et al., 2015; GOG, 2022).

By March 2022, 13 ATMP had received European central MA via the EMA. 2 of them have 2 licensed indications with European central MA (Supplementary Annex S1), making for a total of 15 therapy-indication pairs. All were included in our survey.

Data collection sheets were pre-filled with information from the literature review or previous PPRI Network enquiries where available. Respondents were allowed approximately 3 weeks to respond, with one reminder, and were contacted again to clarify responses that were unclear. The survey included questions about the regulatory approval status in the country (not all operated through the European centralized MA procedure), reimbursement status, the reasons for not reimbursing in case the ATMP is not reimbursed, whether any special arrangements are in place to finance the therapy (such as coverage with evidence development, discounts or rebates–see Supplementary Annex S3 for definitions), the main purpose of special arrangements (for example, control expenditure, share risk), whether information on the scheme is publicly available, how further evidence is to be collected (if any), whether reassessment of the evidence, coverage or price is planned, and any other further information respondents may want to provide. The survey and responses were all in English (the questions asked in the survey are transcribed in Supplementary Annex S4). We reviewed targeted peer reviewed and grey literature to contrast the answers to our survey, and to contextualize them. A draft of this manuscript was circulated amongst responders to ensure we captured their responses accurately. Our focus was on national policies. Within some countries, the manufacturer can negotiate contracts with individual social health insurance bodies, regional health authorities, hospitals, or the private healthcare sector, including P4P schemes. We indicate the cases where our respondent had knowledge of these decentralized agreements, but there may be other similar cases which we were not informed about. We provide a narrative description of results for each country, and consider common themes and suggest policy recommendations in the discussion. The data are anonymized in accordance with the World Health Organization’s (WHO) Framework for Engagement with non-State actors so as not to confer any endorsement of a specific non-State actor’s name, brand or product.

## 3 Results

Responses were received from 20 countries out of 46 (43.5%) (Supplementary Annex S2). 6 of those countries (Armenia, Australia, Brazil, Canada, Israel and Türkiye) do not operate through the European MA procedure (See Supplementary Annex S5). Differences in regulatory status in these countries compared to the EMA, for the ATMPs under study, were observed in 44 instances. The regulatory status in Türkiye, where none of the ATMPs had received regulatory approval at the time of the survey (see Supplementary Table S1 and Supplementary Annex S5 for further details), showed the starkest difference compared to their status with regards to the European centralized regulatory system. Armenia, Brazil, Bulgaria, Iceland, Malta and Türkiye did not reimburse any ATMP (Supplementary Table S1). Malta and Iceland do operate through the European centralized regulatory system, but had not received applications for reimbursement for any ATMPs. To overcome this situation, the government of Malta has an agreement for hematology patients in need of an ATMP to be treated in the United Kingdom. In Brazil, ATMP12 is under assessment and pending a reimbursement decision, for ATMP5 the price has been appealed and ATMP7 was rejected for reimbursement based on the budget impact. Bulgaria, supporting their decision by HTAs in some cases, decided not to fund any of the ATMPs in the list. Armenia gave no reasons for the lack of reimbursement for all ATMPs included in our study, hence we excluded this country from Supplementary Table S1.

14 countries reimbursed at least one ATMP (Supplementary Table S2). Austria and Israel provided no information about P&R schemes. ATMP13 was withdrawn by the manufacturer from Europe. Hence, we did not include it in Supplementary Table S2.

4 of the ATMPs included in our study were chimeric antigen receptors (CAR) T-cells medicines (CAR-Ts) (ATMPs 1, 5, 10 and 11). Previous research in a smaller sample of countries (Germany, Italy, Spain, France and United Kingdom) and ATMPs (11 included, of which 2 were CAR-Ts) found that the CAR-Ts they included in their study were being reimbursed in the countries they observed (Ronco et al., 2021). Our results show wide variation in access across countries for CAR-Ts, with ATMP1 being reimbursed in 2 countries (France and Germany), ATMP5 [indication 5 (I5)] in 13 countries, ATMP5 (indication 6) in 11 countries, ATMP 10 in 4 countries (Israel, France, Germany and Italy), ATMP11 (both for I12 and I13) in the same 11 countries. We observed no systematic differences in reimbursement status (Supplementary Table S1) or P&R arrangement used for reimbursement (Supplementary Table S2) between CAR-Ts and other types of ATMPs.

### 3.1 Australia

In Australia, the purpose of all special arrangements used to finance ATMPs was to share risks. These agreements were always associated to the collection of further evidence. The Pharmaceutical Benefits Advisory Committee (PBAC) does provide advice on the nature of the patient registry that is most suitable in each case (i.e., a disease-based one or therapy-based ones), as well as the minimum data to be collected. For instance, for both indications of ATMP11 and ATMP5, they recommend the Australian Bone Marrow Transplant Recipient Registry, for ATMP7 they recommended including data from Australian patients in the Novartis international registry, and for ATMP12 they noted that a disease-based registry would be suitable, instead of therapy-based registries. For all therapies the manufacturer would be responsible for providing any new data to the HTA committee, which would re-assess the new evidence. The periods for reassessment varied between 2 years from commencement of public financing for both indications of ATMP11 and ATMP5, 3 years for ATMP7 and 5 years for ATMP12.

The special pricing and reimbursement arrangements used for ATMPs were confidential. However, the PBAC does publish its recommendation. For ATMP11, ATMP7 and ATMP12, the PBAC recommended a P4P risk sharing arrangement combined with a confidential discount. For ATMP5, they recommended a P4P.

### 3.2 Canada

In Canada the regulatory authority (Health Products and Food Branch (HPFB) of Health Canada) can issue a Notice of Compliance (NOC), which corresponds to an MA, or a NOC with conditions, corresponding to a Conditional MA. Special agreements to finance medicines are confidential. They may involve simple discounts (e.g., first dollar rebates), incremental rebates in the event an annual threshold is exceeded, and other forms of risk-sharing arrangements. There are special arrangements in place for all 3 ATMPs being reimbursed (ATMP5, ATMP11 and ATMP12). Whether the agreements are linked to the collection of further evidence is also confidential. For therapies that are indeed being subject to the collection of further evidence as part of managed access schemes, such evidence would be meant to inform the clinical and cost-effectiveness parameters of a reassessment (HTA). The institutions responsible for the collection and analysis of this further evidence are the pan-Canadian Pharmaceutical Alliance (pCPA) and/or provincial and territorial drug plans. In Canada, any drug that is reimbursed in the public healthcare system could be eligible for a proactive or reactive reassessment (CADTH, 2022).

### 3.3 Israel

Israel applies special pricing and reimbursement agreements for both indications of both ATMP11 and ATMP5, ATMP2, ATMP7, ATMP10 and ATMP12. However, information about the arrangements is either confidential, not publicly available or not known to the respondents of our survey. In all cases, the schemes are subjects of the collection of further evidence, which is to be collected and analyzed by the Ministry of Health of Israel, although no further information about this is publicly available.

### 3.4 Czechia

In the Czechia, the national HTA body only makes assessments of drugs for outpatient settings. ATMP2 and ATMP3 have been recommended in this context. ATMP2 is subject to a special confidential reimbursement arrangement to control expenditure. ATMP3 is reimbursed without any special arrangement. The HTA body does not assess therapies for in-hospital settings, and have no record of their use. Reimbursement in the hospital settings is theoretically possible for all products within the scope of our study and lies within the competency of health insurance companies and hospitals.

### 3.5 Denmark

Denmark reimburses 4 ATMPs: ATMP3, ATMP7, ATMP12 and ATMP5 (only its indication for B-cell acute lymphoblastic leukemia). ATMP7 is financed by a P4P model in yearly instalments conditioned on continuing clinical response, with data collected by the national procurement agency and healthcare providers (Amgros, 2020). The main aim was to control expenditure.

### 3.6 France

France reimburses most ATMPs (Supplementary Table S1), with confidential price discounts. The information about whether or not the reimbursement arrangements include mandatory evidence collection is confidential. If such data collection was mandated, the responsibility for collecting this information would fall under the Technical Agency for Information on Hospitalization (AITH), and the health ministry would be responsible for analyzing the data. Health technology re-assessment of ATMP11 (both indications), ATMP5 (both indications) and ATMP10 are planned for mid-2023, and in 2024 for ATMP2 and ATMP7. In each case the price can be revised during the entire life cycle of the product. If the HTA assessment indicates that the therapy provides major added clinical value, France has a system to inject additional funding to cover the costs of ATMPs administered in hospitals, on top of the existing diagnosis related group (DRG) fee (Ronco et al., 2021). Eligibility for inclusion in this “add-on list” is based on the cost of the product compared with the tariff applied to the DRG (cost>30% of the tariff). As a result, for ATMP5 and ATMP11, an additional 15,000€ was added in France on top of the DRG fee (Ronco et al., 2021). ATMP3 and ATMP1 were assessed as providing minor added clinical value and no added clinical value respectively, compared with existing alternatives, and so hospitals can use these therapies but receive no additional DRG-funding from the national health insurance system for doing so.

### 3.7 Germany

All ATMPs in this study were being reimbursed in Germany (Schaefer et al., 2021), except for 2 (i.e., ATMP9 and ATMP13), which had been taken off the market by the company (Qiu et al., 2022). In the German market, all new therapies used to be reimbursed at a price freely set by the company during the first year, after which manufacturers negotiate the price of their product with the social insurance providers (Epstein and Espín, 2020). In November 2022, a policy reform (namely, the GKV-Finanzstabilisierungsgesetz or SHI Financial Stabilization Act) shortened the period of free pricing to 6 months (Kleining et al., 2023). In a regular benefit assessment, a drug would only be able to command a premium price if the evidence established a “major” or “substantial” added benefit. The law makes an exception for orphan drugs. Added benefit is “assumed” for orphan drugs as soon as they get European central MA if the total expenditure is less than €50 million per year (Schaefer et al., 2021). Hence in these cases the drugs are reimbursed at premium prices. This has proved controversial (IQWiG, 2022) and concerns have been raised about the spill-over effect on the prices of orphan drugs throughout international markets, since prices of medicines in Germany weigh heavily in the baskets used to estimate reference prices in other countries (Kanavos et al., 2017; Gill et al., 2019). Diverse local MEAs and P4P schemes have been negotiated between the manufacturer and local payers in Germany (Europe, 2019). At the end of 2019 routine practice data collection was required binding the manufacturer to set up a patient registry and to submit results yearly (Benazet et al., 2020; Senior, 2021). In Germany, there are no special arrangements at national level to finance ATMPs (as stated in Supplementary Table S2), but social health insurers negotiate outcomes-based rebates with manufacturers (Jørgensen and Kefalas, 2021; Ronco et al., 2021).

### 3.8 Greece

Greece applies confidential special arrangements to finance ATMP11 (indications 12 and 13) and ATMP5 (indications 5 and 6), ATMP12 and ATMP7. The main aim of the special arrangements is to control expenditure. For ATMP11 and ATMP5 there is a budget cap (there may be additional, confidential, components), with additional data collection over 2 years, followed by a planned reassessment and renegotiation.

### 3.9 Italy

At the time of writing, Italy had decided to reimburse 8 of the ATMPs included in our study, for 10 different indications. To reimburse them, Italy uses a range of types of P&R arrangements (see Supplementary Table S2). Most of the arrangements in place to finance ATMPs in Italy are P4P payment models, paid in instalments (upon result), linked to individual patient data, and applying a confidential discount. Although the size of the discount is kept confidential, information about the P&R arrangement applied is made publicly available in Italy. ATMP7 is reimbursed applying a budget cap, and outcomes are followed through the Italian regulator’s (AIFA) registry (linking prescriptions and payments/rebates to clinical outcomes (Jørgensen et al., 2019)). For ATMP10 and ATMP6, the arrangement is similar but a simple discount was applied instead of a budget cap.

All ATMPs reimbursed in Italy are subject to the collection of further evidence collected by AIFA registries. The technological architecture of the registries is resourced by companies but governed by AIFA (Xoxi et al., 2021). This evidence is subsequently used to reassess the value of the therapy, which usually occurs after 2 years from the agreement signature or in case of extension of indication. Some of these ATMPs were assigned the so called AIFA innovativeness recognition (i.e., ATMP3, ATMP7, ATMP10, ATMP6 and ATMP12), which entitles them to being financed in Italy through a special innovative drug fund, plus becoming immediately available in regional formularies, and exempt from the usual pay-back mechanism (Fortinguerra et al., 2020).

### 3.10 Netherlands (Kingdom of the)

The special arrangements to finance ATMPs are confidential in nature, but in general terms, they were implemented to improve cost-effectiveness and to control expenditures. Only 2 of the special arrangements in place to finance ATMPs in the Netherlands (Kingdom of the) were organized centrally by the government (ATMP7 and ATMP12). The rest were arranged by insurance providers. ATMP11 was re-evaluated based on 3-year survival data and budget impact, which resulted in a confidential discount of the price of at least 5%. Netherlands (Kingdom of the) is also a member of the BENELUXA Initiative, which recently published an HTA jointly produced between the Netherlands (Kingdom of the), Ireland and Belgium for ATMP6 (Policy, 2022), resulting in a recommendation not to reimburse unless cost effectiveness can be improved relative to existing treatment. The countries that constitute the initiative have not yet entered in joint negotiations to reach reimbursement terms for this product (Policy, 2022).

### 3.11 Slovenia

Slovenia applies special arrangements for the reimbursement of ATMP5 (indication 5 and 6), ATMP2 and ATMP12. The main purpose of these financing schemes is to control expenditures, and they achieved this through confidential discounts. None of these schemes are associated with the collection of further evidence.

### 3.12 Spain

In Spain, the special arrangements to finance ATMPs aimed to share risk and to control expenditure. In most cases this comprised a P4P scheme, combined with restrictions in the eligible patient populations. ATMP7 and ATMP12 were financed with P4P schemes combined with expenditure cap and a price-volume agreement respectively. All of them involved the collection of further evidence, which was in all cases operationalized through a national registry operated by the health ministry (Sistema de Información para determinar el VALor TERapéutico de MEDicamentos, which stands for Information System to determine the Therapeutic Value of Medicines, or VALTERMED) (Jørgensen et al., 2020). VALTERMED’s data collection protocols are made publicly available at the website of the Spanish Ministry of Health (both in Spanish and in English). Each decentralized region in Spain has a monitoring committee responsible for data collection and quality. Data analysis and re-assessment will be conducted by the health ministry “when sufficient data become available”, and some provisional data have been published (Sanidad, 2022).

### 3.13 Sweden

In Sweden, the county councils are responsible for in-patient care, which includes ATMPs. A committee called the New Therapies Council supports county councils, enabling the equality of the system. Also, upon request of the regions, the national HTA agency can perform an assessment of the health economic evidence. This level of fragmentation makes it difficult to access information about what financing schemes are in place in Sweden for ATMPs and how they are operationalized. Nevertheless, county councils do publish information about which therapies have a managed entry agreement in place, and the dates associated with reassessment.

Considering the above, although limited in scope, we do have some information about the reimbursement status of ATMPs in Sweden and how it has been operationalized. ATMP11 (indications 12 and 13), ATMP5 (indication 5 only) and ATMP12 are financed through special arrangements. For ATMP11 (indications 12 and 13), a rebate may be required conditional on further evidence collection through the European Society for Blood and Marrow Transplantation (EBMT) patient register and quality local registers. The same registry is used to collect further evidence for ATMP5, but there is no further detail available around the financing arrangement. For ATMP12, the agreement consists of a confidential discount, and the collection of further evidence, operationalized through the national quality register for neuromuscular diseases (NMiS). ATMP7 is the only ATMP reimbursed in Sweden for which there is no public report of a special financing arrangement being in place.

## 4 Discussion

Six countries in this survey reimbursed no ATMPs due to a variety of reasons, including regulatory and reimbursement decisions made by the regulators, the payers or the companies themselves (see Supplementary Table S1 for further details). Where a particular ATMP was financed, there was considerable variability across countries in the types of P&R arrangements used (see Supplementary Table S2 for further details). For instance, ATMP5 and ATMP11 were reimbursed using at least 6 different formulas comprising combinations of P4P, discounts, expenditure caps and restrictions on the patient population. No countries used subscription models or more exotic financial instruments (models and instruments that are further described in Supplementary Annex S3 and discussed in the academic literature (Vogler, 2022b)).

There was considerable variation in the type of P4P schemes for ATMPs in our sample. We identified areas where examples of best practice can be helpful for schemes to achieve their objectives. These included the provision of clear objectives, sharing of information between different departments of the health system, availability of information about the parameters of the agreement (or even whether one exists), and clarity about when, how or by whom the data will be analyzed and re-assessed. Improvement in these areas is a prerequisite that enables the necessary alignment between key stakeholders, including industry and health system actors, for these kinds of schemes to successfully fulfil their purpose, but the necessary human resources and expertise needs to be invested by all involved parties into reaching excellence and productive cross-stakeholder collaboration (Dunlop et al., 2018).

P4P databases in our sample were usually set up using either existing disease registries or purpose-build stand-alone platforms. None of the responses received indicated that routine healthcare administrative databases were used. This may be because, for example, such platforms do not collect the appropriate diagnosis, treatment or outcome variables. The new regulation on European cooperation on HTA does not have any provision for collaboration on post-launch evidence generation (PLEG) (Puñal-Riobóo et al., 2022). This would have enabled the development of common protocols and standards (Iorio et al., 2018; COMET, 2022). The requirement for busy clinicians to manually input (or re-input) P4P data in stand-alone platforms can mean that data is often omitted or duplicated (Ferrario et al., 2017; Godman et al., 2018; Hanna et al., 2018; Michelsen et al., 2020; Facey et al., 2021; Jørgensen and Kefalas, 2021). European cooperation on this area should not only be limited to the actual collection of data, but also on developing capacity in countries, and a further understanding and guiding countries around the methods to quantify the costs and the benefits of risk-sharing, and of the implementation of the different types of schemes available to articulate it (Towse and Garrison, 2010).

At a European level, data sharing across jurisdictions may be essential to leverage the benefits of further evidence generation, especially for ultra-rare diseases (Facey et al., 2021). The role of the European Commission in incentivizing or enforcing the collection of further evidence after conditional centralized marketing authorizations are granted is controversial. Furthermore, research has raised concerns about the delays in the delivery and flaws in the design of post-marketing studies under these schemes, both in Europe and the United States (Salcher-Konrad et al., 2020). The EU has initiated a flagship program to share reports and analyses of regulatory healthcare data (Data Analysis and Real-World Interrogation Network, DARWIN) (Facey et al., 2020). However, perhaps the absence of a central European HTA process and payment mechanism explains that no similar EU-wide initiative addresses the sharing of data that might help address uncertainties at this level, which is a national competency. Furthermore, national governments are responsible for primary data quality. Databases require financial investment (Jørgensen and Kefalas, 2019) and the expertise and leadership to make sure the data is relevant and of sufficient quality (Vogler, 2022b). P4P arrangements can be associated with increased burden to those administering them, while rebates, discounts, price caps and price-volume arrangements can be managed with relatively straightforward contracts and routine administrative healthcare information systems (Hanna et al., 2018). The research undertaken for this paper indicates that there is scope for further European collaboration exploring strategies for countries to build capacity to administer and/or share the burden of the more complex P&R options and increase transparency.

At a country level, the United Kingdom (England) created the Innovative Medicines Fund to ensure fast, provisional access to promising but uncertain treatments, particularly ATMPs, while further evidence is generated (Anderson et al., 2022) and control over budget impact is maintained. The aim of this fund is to provide the system with a route to provide access to selected therapies deemed particularly promising whilst facilitating the collection of further evidence likely to mitigate initial decision uncertainties to avoid the potential opportunity costs associated with these costly therapies (Angelis et al., 2023). The fine details around how this fund is operationalized, particularly around (but not limited to) providing finer definitions of entry requirements such as what is considered to be a promising treatment, or what is deemed to be a ‘step-change in treatment’, and other operational aspects such as what provisions will be put in place for therapies that fail to prove their added value and/or being appropriate use of limited public resources, will determine its success (Angelis et al., 2023). Other countries, such as Italy (Masini et al., 2021) and Canada (Chan et al., 2020; Dai et al., 2021), have developed similar frameworks. Dedicated funds such as these are intended to prevent innovative but uncertain high-cost medicines from displacing other cost-effective interventions while further evidence is generated. However, these siloed funds fragment the pharmaceutical budget and need to be carefully managed and combined with other policies to ensure spending in pharmaceuticals remain affordable and efficient (Mills and Kanavos, 2020). An alternative approach is applied in Australia, where the PBAC has recommended existing disease registries for P4P monitoring. The advantage in principle of disease registries over intervention registries is the potential to estimate comparative effectiveness, subject to appropriate adjustment for confounding by indication (Hatswell et al., 2020). Countries without a defined strategy to fund and manage the collection of further evidence in the context of managed entry agreements might tend to seek simpler P&R agreements with MA holders (such as straight discounts), not because that is the most suitable option to meet their needs in a given P&R decision, but for practicality.

In the sample of responses received, information about the price or the P&R arrangement used to fund a therapy tended to be confidential in nature. While a degree of confidentiality can facilitate negotiation (Joosse et al., 2022), ethically there is a case for enabling reporting of clinical evidence that is accrued using public money under the access schemes (Dal-Ré, 2015; Guerra-Júnior et al., 2017). The World Health Assembly Resolution 72.8 calls for more transparency across a number of areas including prices in other countries, costs of research and patent expiry (Perehudoff, 2022). More transparency across these areas, including MEA schemes, would facilitate P&R decisions and potentially improve access for patients (Commission, 2020; Vogler, 2022a; Webb et al., 2022).

There appears to be considerable variation across regulatory body outcomes. For example, Türkiye has not approved any ATMP and other regulatory bodies have yet to assess all the products. The individual companies choose whether to submit products for regulation and registration in particular countries. For example, whilst a product may have a central European authorization the companies can then decide when and where to launch or file for reimbursement. Our survey shows that the variability of access is in part due to choices made by regulatory and reimbursement authorities, and in part due to commercial decisions by companies about regulatory and reimbursement submissions.

The new European regulation on HTA will help shape the a landscape for ATMPs in the EU, since it stipulates that from 2025 onwards, ATMPs will be required to undergo joint clinical assessments, with the potential of significantly mitigating current differences between national comparative effectiveness assessments (Julian et al., 2022; Angelillo et al., 2023). However, launching and filing for reimbursement and funding decisions will remain at a national level so the overall impact is difficult to assess at this stage. Furthermore, an additional factor that can lead to fragmentation of the EU market is related to the complex manufacturing, logistics and clinical protocols that commercial ATMPs can require (Aguilera-Cobos et al., 2022) and the threat for these costs, or others like the need to translate packaging into each member’s official language, to make smaller countries less commercially attractive for manufacturers, particularly for rare diseases.

The results of our study highlight considerable variation in the approaches used by individual countries to provide access to ATMPs and the scope for voluntary collaborations to overcome some of the existing barriers, particularly for smaller countries. For example, some of the options available to them include joint P&R negotiations for new medicines for demand pooling (to increase the volume), collaboration on the administration of ATMPs (through joint treatment centers), or cross-country collaboration on real-world-evidence generation (Angelillo et al., 2023). There are a number of good examples of collaboration in the European region: FINOSE (Finland, Norway, Sweden), BENELUXA or the Valletta Declaration, or bilateral arrangements such as those between Malta and the United Kingdom (i.e., Malta has an agreement for hematology patients in need of an ATMP to be treated in the United Kingdom, as presented at the beginning of the results section).

The development of detailed treatment protocols (including all associated costs), and clear communication of it to stakeholders, would facilitate cross-border collaboration enabling international multidisciplinary care teams to build on existing infrastructures such as the European Reference Networks (ERNs) to deliver care and to collect evidence, which would provide a European instrument to collaborate towards mitigating uncertainties (Angelillo et al., 2023). The view of patient representatives is that, although pooled procurement of ATMPs has not yet been extensively explored, it should be considered more widely (Benvenuti et al., 2021). Options suggested to boost cross-border collaboration in Europe to enhance access to ATMPs include innovative solutions that are yet to be tried, such as providing care through regional expert treatment centers (Angelillo et al., 2023).

As the evidence we present in this paper shows, many products are not submitted for reimbursement in individual countries with priority being given to larger markets. Members of the European Federation of Pharmaceutical Industries and Associations (EFPIA) have committed to “file for pricing and reimbursement in all EU countries as soon as possible and no later than 2 years from the central EU market authorization, provided that local systems allow it” (Associations, 2022). The Pharmaceutical Strategy for Europe notes that many developers of ATMPs benefit from financial or other incentives during the development phases and the EC is exploring “conditionality” of those push incentives to support broader access and increase competition (Commission, 2020). However these proposals have sparked significant debate and reactions from stakeholders, including representatives of the Commission (Gallina, 2023), hospital pharmacists representatives (Kohl, 2021), the European pharmaceutical industry (Associations, 2020) and academic researchers (Garattini et al., 2021) amongst others. There is considerable variation in ability to pay across the European Region. Therefore, in order to support equitable access across smaller and lower income countries, more explicit consideration of pricing principles will be required, ensuring that any use of external reference pricing is appropriate and mechanisms to preventing arbitrage are in place (Docteur, 2022).

Our survey has only included “commercial” ATMPs, developed by private MA holders. There are also now several so-called “academic” ATMPs (Egea-Guerrero et al., 2019; Juan et al., 2021; Trias et al., 2022), developed by non-profits (EMA, 2022b) or public-private collaborations (Priesner and Hildebrandt, 2022) under hospital exemption regulations (Coppens et al., 2020; Trias et al., 2022). In some cases the manufacturer is preparing for centralized MA (EMA, 2022b). The potential role of academic ATMPs has been highlighted as a potential route to creating a generic market for this kind of therapies, however multiple barriers prevent this from happening (Seoane-Vazquez et al., 2019). It remains to be seen how regulation, pricing and competitiveness of academic ATMPs will compare with commercial ones (Cuende et al., 2014; Seoane-Vazquez et al., 2019).

### 4.1 Strengths and limitations of this study

This paper has described the P&R landscape in 2022 for 15 ATMPs in 20 countries, a much larger sample of products and countries than other articles (Jørgensen et al., 2020; Jørgensen and Kefalas, 2021; Ronco et al., 2021). There may of course be other arrangements in other countries. The countries were mainly high-income, with two upper middle-income. More research is needed on P&R arrangements in low- and middle-income countries (Castro et al., 2019), and in smaller countries too (focusing for instance in the countries included in the WHO led Small Countries Initiative–a network of 11 European countries with 2 million or less inhabitants, out of which 3 were included in our survey). The survey was in English, which was not the first language of most respondents. We attempted to clarify and classify common terms with respondents across diverse language and institutional settings. The survey was directed at national authorities for P&R. To greater or lesser extent, decision making may be decentralized, as in Sweden, Germany and Spain.

## 5 Conclusions and recommendations

In this section, and in Supplementary Table S3, we have summarized the key areas for further development and the recommendations associated to each.

The work undertaken has demonstrated that there is wide variation in access to ATMPs between the countries surveyed. Furthermore, that this variation has a number of reasons including regulatory differences, commercial decisions by MA holders, and the divergent assessment processes and criteria applied by payers. Moving towards greater equality of access will require cooperation between countries and stakeholders, together with relevant international actors such as the WHO Regional Office for Europe’s Access to Novel Medicines Platform.

There is also considerable cross-country variation in how P4P schemes are used for a particular ATMPs. This imposes transaction costs on healthcare systems and MA holders, and limits opportunity for data sharing. In line with WHA 72.8, greater transparency, particularly where public funding has been used, will enable dialogue about the schemes in use, and the development of common protocols, terminology and standards for data collection, will lower costs and generate better quality evidence, ultimately with benefits for patients.

The inclusion of post-launch evidence generation in the new European regulation on cooperation in HTA could formalize arrangements. A specific proposal along these lines was made by EURORDIS, which suggested the co-creation, with multi-stakeholder input, of a data strategy for the European Reference Networks (ERNs) to progress towards the common implementation of a European data infrastructure, building on the existing infrastructure of the Networks (EURORDIS, 2020).

Demand pooling and pooled procurement of ATMPs has not yet been frequently used, should be considered more widely (Benvenuti et al., 2021) and could facilitate evaluation, evidence generation, pricing and ultimately access in all countries due to the stronger negotiating position they would acquire, but particularly in small countries (Angelillo et al., 2023).

There have been several examples of non-profit development of “academic” ATMPs. Careful evaluation of these initiatives should be undertaken, considering the legal and regulatory framework, accounting methods for estimating costs, incentives, P&R pathways for these kinds of products and the implications for competition with commercial medicines.

In the mid-term, more investment in enhancing HTA and (other) infrastructures to support P&R processes (be it through a strong European HTA infrastructure supporting the new regulation, and/or enhancing resources deployed nationally), accompanied by coordinated efforts to further develop the necessary expertise, would highly benefit decision makers dealing with complex P&R decisions for ATMPs.

## Data Availability

The datasets presented in this article are not readily available because The data are anonymized in accordance with the World Health Organization’s (WHO) framework for engagement with non-State actors so as not to confer any endorsement of a specific non-State actor’s name, brand or product. Requests to access the datasets should be directed to SG, garners@who.int.
