# Supplementary material for: Pricing and reimbursement mechanisms for advanced therapy medicinal products in 20 countries
Source: Front Pharmacol. 2023 Nov 28;14:1199500. doi: 10.3389/fphar.2023.1199500 (PMC10715052; doi:10.3389/fphar.2023.1199500)
Supplement: Supplementary file 1 [file Table1.docx]

Supplementary Material

Pricing and Reimbursement Mechanisms for Advanced Therapeutic Medicinal Products in 20 countries

Juan Carlos Rejon-Parrilla*, Jaime Espín, Sarah Garner, Stanislav Kniazkov, David Epstein

*** Correspondence:** Juan Carlos Rejon-Parrilla: juancarlos.rejon@juntadeandalucia.es

In this document, below, we present Tables 1, 2 and 3 and Annexes I, II, III, IV, V and VI. They are all mentioned in the body of the paper.

**Table 1. Reimbursement status and reasons for not financing ATMPs**

|  | | ***Reimbursement status and reasons for not financing per country*** | | | | | | | | | | | | |
| --- | --- | --- | --- | --- | --- | --- | --- | --- | --- | --- | --- | --- | --- | --- |
|  | | ***Countries with national MA only*** | | | | | ***Countries that*** ***operate through the European MA procedure*** | | | | | | | |
| ***Countries*** | | ***Australia*** | ***Canada*** | ***Israel*** | ***Brazil*** | ***Turkey*** | ***Austria*** | ***Bulgaria*** | ***Czechia*** | ***Denmark*** | ***France*** | ***Germany*** | ***Greece*** |  |
| ***ATMP*** | ***Indication*** |  |  |  |  |  |  |  |  |  |  |  |  |  |
| ***ATMP1*** | ***MM(I1)*** | NRA | O | NRA | NRA | NRA | NRD | NSR | NSR^a^ | O | R^c^ | R | NSR |  |
| ***ATMP2*** | ***AFCDP(I2)*** | NRA | NRA | R | NRA | NRA | R | NSR | R^a^ | NRD | R | R | NRD |  |
| ***ATMP3*** | ***RDCC(I3)*** | NRA | NRA | NRA | NRA | NRA | NRD | NSR | R^a^ | R | R^c^ | R | NSR |  |
| ***ATMP4*** | ***MNRBS(I4)*** | NRA | NRA | NRA | NRA | NRA | NRD | NSR | NSR^a^ | O | NSR | R | NSR |  |
| ***ATMP5*** | ***BCALL(I5)*** | R | R | R | O | NRA | R | NSR | NSR^a^ | R | R | R | R |  |
| ***ATMP5*** | ***DLBCL(I6)*** | R | R | R | O | NRA | R | NSR | NSR^a^ | NRD | R | R | R |  |
| ***ATMP6*** | ***ML(I7)*** | NRA | NRA | NRA | NRA | NRA | NRD | NSR | NSR^a^ | R | O | R | NSR |  |
| ***ATMP7*** | ***HRD(I8)*** | R | O | R | NRD | NRA | R | NSR | NSR^a^ | R | R | R | R |  |
| ***ATMP8*** | ***KCR(I9)*** | NRA | NRA | NRA | NRA | NRA | NRD | NSR | NSR^a^ | O | NRD | R | NSR |  |
| ***ATMP9*** | ***SCIDTADD (I10)*** | NRA | NRA | NRA | NRA | NRA | NRD | NSR | NSR^a^ | O | NSR | NRA^d^ | NSR |  |
| ***ATMP10*** | ***MCL(I11)*** | O | O | R | NRA | NRA | NRD | NSR | NSR^a^ | O | R | R | O |  |
| ***ATMP11*** | ***DLBCL(I12)*** | R | R | R | NRA | NRA | R | NSR | NSR^a^ | NRD^b^ / O^b^ | R | R | R |  |
| ***ATMP11*** | ***PML(I13)*** | R | R | R | NRA | NRA | R | NSR | NSR^a^ | O | R | R | R |  |
| ***ATMP12*** | ***SMA(I14)*** | R | R | R | O | NRA | R | O | NSR^a^ | R | O | R | R |  |
| ***ATMP13 ^e^*** | ***BT(I15)*** | NRA | NRA | NRA | NRA | NRA | NRD | NSR | NSR^a^ | NRA | NRA | NRA | NRA |  |

Legend: See Annex VI for indication abbreviations; MA = Marketing authorization; NRA = No regulatory approval or withdrawn at the request of the manufacturer; NRD = Negative reimbursement decision or reason for not reimbursement not reported in response to our survey; NSR = No submission received for public reimbursement; O = Ongoing (includes awaiting or ongoing health technology assessment, or assessment done but no reimbursement decision made); R = Reimbursed.

Note: ^a^ In the Czechia, the SÚKL (State Institute for Drug Control – the institution tasked with supporting the regulation of prices and reimbursements for pharmaceuticals) does not participate in price-setting or reimbursement in the in-hospital setting. They do however do so for out-patient therapies (see Results for Czechia). ^b^ In Denmark, ATMP11 for DLBCL has been assessed and not recommended for third line treatment, and an application for assessment for second line treatment has been put forward (assessment yet to be done); ^c^ In France, ATMP1 and ATMP3 are reimbursed, but the reimbursement payment to the hospital is no greater than that for standard care. ^d^ ATMP9 was withdrawn by the manufacturer in Germany; ^e^ ATMP13 was withdrawn by the manufacturer from Europe. Armenia did not give reasons for non-reimbursement and is not included in Table 1.

(continues below)

(continuation of table 1)

|  | | ***Reimbursement status and reasons for not financing per country*** | | | | | | |
| --- | --- | --- | --- | --- | --- | --- | --- | --- |
|  | | ***Countries that operate through the European MA procedure*** | | | | | | |
| ***Countries*** | | ***Iceland*** | ***Italy*** | ***Malta*** | ***Netherlands (Kingdom of the)*** | ***Slovenia*** | ***Spain*** | ***Sweden*** |
| ***ATMP*** | ***Indication*** |  |  |  |  |  |  |  |
| ***ATMP1*** | ***MM(I1)*** | NSR | O | NSR | O | NSR | O | O |
| ***ATMP2*** | ***AFCDP(I2)*** | NSR | NRD | NSR | R | R | R | NRD |
| ***ATMP3*** | ***RDCC(I3)*** | NSR | R | NSR | R | NSR | NRD | NRD |
| ***ATMP4*** | ***MNRBS(I4)*** | NSR | NSR | NSR | R | NSR | NRD | NRD |
| ***ATMP5*** | ***BCALL(I5)*** | NSR | R | NSR | R | R | R | R |
| ***ATMP5*** | ***DLBCL(I6)*** | NSR | R | NSR | R | R | R | NRD |
| ***ATMP6*** | ***ML(I7)*** | NSR | R | NSR | O | NSR | O | O |
| ***ATMP7*** | ***HRD(I8)*** | NSR | R | NSR | R | NSR | R | R |
| ***ATMP8*** | ***KCR(I9)*** | NSR | NRD | NSR | R | NSR | NSR | NRD |
| ***ATMP9*** | ***SCIDTADD(I10)*** | NSR | R | NSR | R | NSR | NSR | O |
| ***ATMP10*** | ***MCL(I11)*** | NSR | R | NSR | O | NSR | NRD | NRD |
| ***ATMP11*** | ***DLBCL(I12)*** | NSR | R | NSR | R | NSR | R | R |
| ***ATMP11*** | ***PML(I13)*** | NSR | R | NSR | R | NSR | R | R |
| ***ATMP12*** | ***SMA(I14)*** | NSR | R | NSR | R | R | R | R |
| ***ATMP13*** | ***BT(I15)*** | NSR | NRA | NSR | NRA | NRA | NRA | NRA |

Legend: See Annex VI for indication abbreviations; MA = Marketing authorization; NRA = No regulatory approval or withdrawn at the request of the manufacturer; NRD = Negative reimbursement decision or reason for not reimbursement not reported in response to our survey; NSR = No submission received for public reimbursement; O = Ongoing (includes ongoing assessment, assessment not yet done and assessment done but no reimbursement decision made); R = Reimbursed.

**Table 2.** **P&R arrangements, negotiated at national level between manufacturers and official national institutions, for reimbursed ATMPs in 12 countries**

|  | | ***Types of MEAs used to finance ATMPs and purpose in 12 countries*** | | | | | | | | | | | |
| --- | --- | --- | --- | --- | --- | --- | --- | --- | --- | --- | --- | --- | --- |
|  | | ***Countries with national MA only*** | | | | ***Countries that operate through the European MA procedure*** | | | | | | | |
| ***Countries*** | | ***Australia*** | | ***Canada*** | | ***Czechia*** | | ***Denmark*** | | ***France*** | | ***Germany*** | |
| ***ATMP*** | ***Indication*** | ***MEA*** | ***Purpose*** | ***MEA*** | ***Purpose*** | ***MEA*** | ***Purpose*** | ***MEA*** | ***Purpose*** | ***MEA*** | ***Purpose*** | ***MEA*** | ***Purpose*** |
| ***ATMP1*** | ***MM*** | NR | / | NR | / | NR | / | NR | / | NR | / | R | / |
| ***ATMP2*** | ***AFCDP*** | NR | / | NR | / | D | CE | NR | / | D | CE | D | NA |
| ***ATMP3*** | ***RDCC*** | NR | / | NR | / | R | / | R | / | NR | / | R | / |
| ***ATMP4*** | ***MNRBS*** | NR | / | NR | / | NR | / | NR | / | NR | / | R | / |
| ***ATMP5*** | ***BCALL*** | P4P | SR | C | CE; SR* | NR | / | R | / | D | CE | CED; P4P; D | NA |
| ***ATMP5*** | ***DLBCL*** | P4P | SR | C | CE; SR* | NR | / | NR | / | D | CE | CED; P4P; D | NA |
| ***ATMP6*** | ***ML*** | NR | / | NR | / | NR | / | R* | / | NR | / | R | / |
| ***ATMP7*** | ***HRD*** | D; P4P | SR | NR | / | NR | / | P4Pi | CE | D | CE | CED; D | NA |
| ***ATMP8*** | ***KCR*** | NR | / | NR | / | NR | / | NR | / | NR | / | R | / |
| ***ATMP9*** | ***SCIDTADD*** | NR | / | NR | / | NR | / | NR | / | NR | / | NR | / |
| ***ATMP10*** | ***MCL*** | RNI | RNI | NR | / | NR | / | NR | / | D | CE | R | / |
| ***ATMP11*** | ***DLBCL*** | P4P; D | SR | C | CE; SR* | NR | / | NR | / | D | CE | CED; P4P; D | NA |
| ***ATMP11*** | ***PML*** | P4P; D | SR | C | CE; SR* | NR | / | NR | / | D | CE | CED; P4P; D | NA |
| ***ATMP12*** | ***SMA*** | P4P; D | SR | C | CE; SR* | NR | / | R | / | NR | / | P4Pi | NA |

Legend: See Annex VI for indication abbreviations; C = Confidential; CE = Control Expenditure; CED = Coverage with Evidence Development; D = Discount; MA = Marketing authorization; NA = information Not Available; NR = Not Reimbursed; P4P = Pay-for-performance; P4Pi = P4P in instalments; R = Reimbursed without using any special arrangements; RNI = Reimbursement decision published but Not yet Implemented; SR = Share Risk.

* Note: the nature of agreements in Canada is confidential. They may involve simple discounts (e.g. first dollar rebates), incremental rebates in the event an annual threshold is exceeded, and other forms of risk-sharing arrangements; ATMP6 is reimbursed by the Danish healthcare system, but it is not delivered in Denmark. Hence, the Danish Medicines Council (DMC) – the institution responsible for assessing the clinical value of new medicines and new indications in Denmark – will not assess this treatment.

(continues below)

(continuation of table 2)

|  | | ***Types of MEAs used to finance ATMPs and purpose in 12 countries*** | | | | | | | | | | | |
| --- | --- | --- | --- | --- | --- | --- | --- | --- | --- | --- | --- | --- | --- |
|  | | ***Countries that operate through the European MA procedure*** | | | | | | | | | | | |
| ***Countries*** | | ***Greece*** | | ***Italy*** | | ***Netherlands (Kingdom of the)*** | | ***Slovenia*** | | ***Spain*** | | ***Sweden*** | |
| ***ATMP*** | ***Indication*** | ***MEA*** | ***Purpose*** | ***MEA*** | ***Purpose*** | ***MEA*** | ***Purpose*** | ***MEA*** | ***Purpose*** | ***MEA*** | ***Purpose*** | ***MEA*** | ***Purpose*** |
| ***ATMP1*** | ***MM*** | NR | / | NR | / | NR | / | NR | / | NR | / | NR | / |
| ***ATMP2*** | ***AFCDP*** | NR | / | NR | / | NA | C* | D | CE | P4P; RPP | CE; SR | NA | NA |
| ***ATMP3*** | ***RDCC*** | NR | / | P4P; D | NA | NA | C* | NR | / | NR | / | NR | / |
| ***ATMP4*** | ***MNRBS*** | NR | / | NR | / | NA | C* | NR | / | NR | / | NR | / |
| ***ATMP5*** | ***BCALL*** | C | CE | P4Pi; D | NA | C | C* | D | CE | P4P; RPP | CE; SR | NR | / |
| ***ATMP5*** | ***DLBCL*** | C | CE | P4Pi; D | NA | C | C* | D | CE | P4P; RPP | CE; SR | NA | NA |
| ***ATMP6*** | ***ML*** | NR | / | D; MR | NA | NR | / | NR | / | NR | / | NR | / |
| ***ATMP7*** | ***HRD*** | C | CE | EC; MR | NA | C | C* | NR | / | P4P; EC | CE; SR | NA | NA |
| ***ATMP8*** | ***KCR*** | NR | / | NR | / | NA | C* | NR | / | NR | / | NR | / |
| ***ATMP9*** | ***SCIDTADD*** | NR | / | P4PIPP; D | NA | NA | C* | NR | / | NR | / | NR | / |
| ***ATMP10*** | ***MCL*** | NR | / | MR; D | NA | NR | / | NR | / | NR | / | NR | / |
| ***ATMP11*** | ***DLBCL*** | C | CE | P4Pi; D | NA | C | C* | NR | / | P4P; RPP | CE; SR | RB | / |
| ***ATMP11*** | ***PML*** | C | CE | P4Pi; D | NA | C | C* | NR | / | P4P; RPP | CE; SR | RB | / |
| ***ATMP12*** | ***SMA*** | C | CE | P4P | NA | C | C* | D | CE | P4P; P-V | CE; SR | D | NA |

Legend: See Annex VI for indication abbreviations; C = Confidential; CE = Control Expenditure; D = Discount; EC = Expenditure Cap; MA = Marketing authorization; MR = Monitored by Registry; NA = information Not Available; NR = Not Reimbursed; P-V = Price-volume arrangement; P4P = Pay-for-performance; P4Pi = P4P in instalments; P4PIPP = P4P Linked to Individual Patient Data; RB = Rebate; RPP = Restricted Patient Population; SR = Share Risk.

* Note: in general, the key aims of any MEAs used in Netherlands (Kingdom of the), as reported by the Dutch responder to our survey, are to improve cost-effectiveness and control expenditure.

Austria and Israel are not included Table 2 because they provided no information on MEAs on confidentiality grounds.

**Table 3. Key areas for further development and associated recommendations**

| **Key challenges** | **Recommendations** |
| --- | --- |
| There is no co-ordination mechanism for RWE to be collected to meet the needs of regulators, HTA agencies and payer organisations purposes | (i) A pan-European approach could be considered for post-launch evidence generation, for example enabled through the new European regulation on cooperation in HTA, in coordination with the implementation of the European data infrastructure and research networks., (ii) More investment and cooperation on capacity building around the implementation of risk sharing schemes and the design and administration of the data collection protocols associated to them, should also be considered |
| There is considerable variation between countries in the reporting of clinical evidence that has been in part funded with public resources under managed entry schemes. Different countries approach the generation of further evidence under these schemes differently. | The World Health Assembly Resolution 72.8 calls for more transparency, across a number of areas, including prices and managed entry schemes. Greater transparency and fluid communication about the schemes in use, and common practices and requirements for data collection, will generate better quality evidence at lower costs, ultimately benefiting patients. |
| There is heterogeneity in regulatory and P&R status of ATMPs across countries. Our survey shows that the variability of access is in part due to choices made by regulatory and reimbursement authorities, and in part due to commercial decisions by companies about regulatory and reimbursement submissions | (i) The new European regulation on HTA will provide more homogeneity in the relative effectiveness evidence used to support national HTA processes. Further cross-country collaboration in the economic evaluation and even in joint procurement of ATMPs could further reduce variability in access, particularly for products with an immature evidence base.  (ii) Further developing HTA and (other) infrastructures to support P&R processes (investing in European HTA infrastructures to sustain the new regulation, and/or increasing national investments), in parallel with coordinated action to building up the necessary expertise, would highly benefit decision makers dealing with complex P&R decisions for ATMPs.  (iii) Fulfilling EFPIA’s commitment for MA holders to file for P&R in all countries within 2 years from central European Union MA would be a great further step toward reducing variabilities in access although not all companies/countries have the capacity to support this. |
| The regulatory environment in Europe is moving towards providing greater support for the development of ATMPs by academic and non-for-profit institutions, but it remains to be seen how the regulatory requirements (under the hospital exemption), pricing and competitiveness of academic ATMPs will compare with commercial ones | Careful evaluation of the regulatory and P&R environments for academic ATMPs, and the implications for competition with commercial medicines, should be undertaken, to ensure safe and effective academic ATMPs that respond to unmet needs are developed, and that they are met with established P&R pathways |
| Demand pooling and joint purchasing has barely been explored for ATMPs | Such approaches could facilitate evaluation, evidence generation, pricing and ultimately access due to the stronger negotiating position countries would acquire. Collaboration could go from joint assessments and/or negotiations, going as far as exploring options for joint European treatment centres. These solutions could particularly benefit smaller countries |
| Overarching recommendation: Moving towards greater equality of access will require cooperation between countries and stakeholders, and there is infrastructure and mechanisms that could facilitate this such as the WHO Regional Office for Europe’s Access to Novel Medicines Platform. | |

| **ANNEX I – EMA regulatory categories applied to ATMPs and whether given in a single administration** |
| --- |

| **ATMP** | **Indication** | **PRIME (unmet need)** | **Orphan** | **Intended for single administration?** | **Marketing authorization (MA) in European Union** | **Date of MA by the European Medicines Agency** |
| --- | --- | --- | --- | --- | --- | --- |
| ATMP1 | MM | Yes | Yes | Yes | Conditional MA | 18/08/2021 |
| ATMP2 | AFCDP | No | Yes | Yes | Additional monitoring | 23/03/2018 |
| ATMP3 | RDCC | No | Yes | Yes | Conditional MA | 17/02/2015 |
| ATMP4 | MNRBS | No | No | No | Standard MA | 16/12/2015 |
| ATMP5 | BCALL | Yes | Yes | Yes | Additional monitoring | 22/08/2018 |
| ATMP5 | DLBCL | No | Yes | Yes | Additional monitoring | 22/08/2018 |
| ATMP6 | ML | No | Yes | Yes | Additional monitoring | 17/12/2020 |
| ATMP7 | HRD | No | Yes | Yes | Additional monitoring | 22/11/2018 |
| ATMP8 | KCR | No | No | Yes | Standard MA | 10/07/2017 |
| ATMP9 | SCIDTADD | No | Yes | Yes | Additional monitoring | 26/05/2016 |
| ATMP10 | MCL | Yes | Yes | Yes | Conditional MA | 14/12/2020 |
| ATMP11 | DLBCL | Yes | Yes | Yes | Additional monitoring | 23/08/2018 |
| ATMP11 | PML | No | Yes | Yes | Additional monitoring | 23/08/2018 |
| ATMP12 | SMA | Yes | Yes | Yes | Conditional MA | 18/05/2020 |
| ATMP13 | BT | Yes | Yes | Yes | Conditional MA | 29/05/2019 |

Legend: See Annex VI for indication abbreviations. The data are anonymized in accordance with the World Health Organization’s (WHO) Framework for Engagement with non-State actors so as not to confer any endorsement of a specific non-State actor’s name, brand or product (See Data Availability statement in the manuscript).

Note: in some cases, the ATMP can be intended for a single administration but allow repetition of treatment if the physician considers it necessary.

| **ANNEX II – List of the 46 countries of origin of PPRI Network members we contacted for our survey, which ones responded and which ones did not respond** |
| --- |

**List of countries of origin of members of the PPRI Network that received the survey (a total of 46)**

Albania, Armenia, Australia, Austria, Belgium, Brazil, Bulgaria, Canada, Croatia, Cyprus, Czechia, Denmark, Egypt, Estonia, Finland, France, Germany, Greece, Hungary, Iceland, Ireland, Israel, Italy, Kosovo^[[1]](#footnote-1)^, Latvia, Lithuania, Malta, Netherlands (Kingdom of the), North Macedonia, Norway, Portugal, Republic of Korea, Republic of Moldova, Republic of Serbia, Romania, Saudi Arabia, Singapore, Slovakia, Slovenia, Spain, Sweden, Switzerland, Türkiye, Ukraine and United Kingdom.

**List of countries of origin of members of the PPRI Network that responded to the survey (a total of 20)**

Outside the EMA regulatory jurisdiction: Armenia, Australia, Brazil, Canada, Israel and Türkiye.

Within the EMA regulatory jurisdiction: Austria, Bulgaria, Czechia, Denmark, France, Germany, Greece, Iceland, Italy, Malta, Netherlands (Kingdom of the), Slovenia, Spain, Sweden.

**List of countries of origin of members of the PPRI Network that did not respond to the survey (a total of 26)**

Albania, Belgium, Croatia, Cyprus, Egypt, Estonia, Finland, Hungary, Ireland, Kosovo^[[2]](#footnote-2)^, Latvia, Lithuania, North Macedonia, Norway, Portugal, Republic of Korea, Republic of Moldova, Republic of Serbia, Romania, Saudi Arabia, Singapore, Slovakia, Switzerland, Ukraine and the United Kingdom.

Note: due to human error, there were 5 discrepancies between the countries of origin of members of the PPRI Network and those we invited to respond to our survey at the time when we distributed the survey. Experts from Luxembourg, Kazakhstan, Kyrgyzstan and South Africa were members of the PPRI Network but they did not receive an invitation to participate in our survey, and there were no Russian members within the PPRI Network, yet one was invited to participate (but did not do so).

| **ANNEX III – Taxonomy of Pricing and Reimbursement options** |
| --- |

| Type of P&R arrangement | Purpose | Counterparties involved | Country experience/s or example | Reference/s |
| --- | --- | --- | --- | --- |
| *Financial agreements* | ***Agreements based on financial aspects, independent of health outcomes achieved*** | ***NA*** | ***NA*** | ***(Hanna et al., 2018)*** |
| Bundle payment, episode of care | A single payment to cover all the care a patient with a condition may need. Aim: to incentivise organisations to control costs, without sacrificing quality, thereby increasing efficiency. | Payers/Insurers (public or private), Service providers | United States | (Hanna et al., 2018, Hussey et al., 2011) |
| Rebate | Payment that is refunded by the manufacturer to the payer if a set of pre-agreed conditions occur. Aim: to lower costs. | Payers/Insurers, Manufacturers | Switzerland | (Gavious et al., 2014, Hanna et al., 2018, Carl and Vokinger, 2021) |
| Discounts | Price reductions (often confidential). Aim: cost containment measure. | Payers/Insurers, Manufacturers | Selection of 25 European countries | (Vogler et al., 2012, Hanna et al., 2018) |
| Price caps and volume caps | Aim: to control prices or total expenditure on a given medicine. They can be implemented on a patient level (capping the yearly price or the number of yearly courses reimbursed) or at a population level (limiting the volume of product to be sold yearly – the manufacturer reimburses the full cost or a fraction) | Payers/Insurers, Manufacturers | Republic of Korea | (Hanna et al., 2018, Lee et al., 2021) |
| Price-volume agreements | The price of the product is adjusted based on volumes sold – the percentage of reduction is pre-agreed. Aim: to reduce prices. | Payers/Insurers, Manufacturers | Denmark, France, Italy, Lithuania, Spain | (Hanna et al., 2018, Andersson et al., 2020, WHO, 2016, Messori, 2016b, Messori, 2016a) |
| Lump-sum or subscription model | Agreement between the payer and the manufacturer to make treatment available for a fixed amount over a period of time. The payment is often spread over such period. Aim: to reduce financial uncertainty, prevent high-upfront costs or facilitate access to large populations. | Payers/Insurers, Manufacturers | Australia, United States | (Moon and Erickson, 2019, Coyle et al., 2020, Vreman et al., 2020, Trusheim et al., 2018) |
| Cost-plus price | Price set according to development and production costs producing a pre-agreed amount fixing the revenues. Proposed for orphan drugs that are not cost-effective, but discouraged by WHO. Aim: cost containment. | Manufacturers, Payers | Sweden, Japan | (Hanna et al., 2018, WHO, 2020, Takayama and Narukawa, 2016, Persson et al., 2012) |
| Drug mortgages, healthcare loans / Credits | Can be offered to payers or patients. Aim: to spread the cost of treatment. | Manufacturers, Payers or Patients | Spain | (Hanna et al., 2018, Hampson et al., 2018, Diego, 2015) |
| Reinsurance risk pool | Multiple payers share risk through a reinsurance risk pool. Aim: to ensure single payers from risks around high costs of treatment. It helps prevent cherry picking in systems with multiple (private) insurers | (Multiple) Payers/Insurers | United States | (Hanna et al., 2018, Zettler and EC, 2017) |
| National silo funds | National funds dedicated to a particular disease or disease area. Aim: to secure funding for the condition/s or type/s of therapy(ies) covered, and can aim to optimise outcomes within a therapeutic area. | Payer/Insurer (national or regional in public systems), Service Provider/s, Manufacturers, can involve (or be managed by) the HTA body | Italy, United Kingdom (England) | (Hanna et al., 2018, Masini et al., 2021, Mills and Kanavos, 2020, Anderson et al., 2022) |
| Special international fund raising | International taxes on specific transactions. Aim: to finance provision of medicines (e.g., the airline ticket levy first implemented by France), pooling of donor aid to create a predictable demand, and waiving debt when the lender country negotiates commitments by the LMIC receiving the loan to dedicate the money saved into the provision of a medical service. | Payer, Other sectors of Government, International Fund (donor aid), third party country that acts as a lender or waiver of debt | France | (Hanna et al., 2018, Meghani and Basu, 2015) |
| Intellectual property-based payment | Includes prizes for patents, either to buy the patent and control production and distribution, or extensions of market exclusivity as a reward for innovation in an area of unmet need (as it happens with the orphan drug designation in the European Union). Aim: incentivize innovation in a priority area. | Payer, Manufacturer, Regulator | European Union | (Hanna et al., 2018, Carr and Bradshaw, 2016, Micallef and Blin, 2020) |
| Tiered pricing | It consists of setting different prices for the same product in different countries, according to their income levels. Aim: to facilitate access in LMICs. | Payers, Manufacturers, International Organisations (e.g., United Nations Children's Fund (UNICEF)) | Africa, India | (Babar and Atif, 2014) |
| *Health outcomes-based agreements* | ***Agreements based on the performance of the new therapy*** |  | ***NA*** | ***(Hanna et al., 2018)*** |
| Pay-for-performance (P4P) | Price or revenue are linked to the performance of the medicine. Aim: incentivise access to medicines that perform highly in clinical practice, share risk, and control budget impact. | Payer, Manufacturer, National Health Service | Bulgaria, France, Germany, Italy, Poland, Romania, Spain, United Kingdom | (Garrison Jr et al., 2013, Hanna et al., 2018, Wenzl and Chapman, 2019, Clopes et al., 2017, Jørgensen and Kefalas, 2021, Jørgensen et al., 2020, Kamusheva et al., 2021) |
| Indication-specific pricing | Links the price to the performance of the medicine in each indication. Aim: prevent companies from focusing on high-performing indications to maintain a high price. | Payer, Manufacturers | Italy | (Preckler and Espín, 2022, Hanna et al., 2018) |
| Rebate risk sharing | The share of co-payment decreases as patients complete cycles of treatment. Aim: incentivise adherence. | Payers/Insurers, Manufacturers, Patients | United States | (Kleinke and McGee, 2015, Hanna et al., 2018) |
| Limit pricing approach | Includes payment for outcomes, where performance targets that are not met entail price reductions. That is, the limit price represents a threshold that, if exceeded, involves a net increase in healthcare expenditures (similar to the NICE threshold). Aim: maximise health outcomes per monetary unit invested. | Payer, Manufacturers, HTA bodies | United States | (Hanna et al., 2018, Fuller and Goldfield, 2016) |
| Annuity payments | Spread the cost of the medicine over a longer period of time (than high upfront payments) when pre-agreed clinical endpoints are met. Aim: manage large upfront costs and share risk. | Payers, Manufacturers | United Kingdom (England), Italy | (Hanna et al., 2018, Jørgensen and Kefalas, 2017, Jørgensen and Kefalas, 2021) |
| Coverage with evidence development (CED) | Conditional reimbursement subject to collection of further evidence in clinical practice, with a reassessment within a pre-agreed timeframe before a final reimbursement and pricing decision is made. Aim: reduce large initial uncertainties and share risk. | Payers, Manufacturers, Service Provider/s | France, Germany, Netherlands (Kingdom of the), Sweden, Switzerland, United Kingdom | (Hanna et al., 2018, Brügger et al., 2014, Dabbous et al., 2020, Jørgensen and Kefalas, 2021) |
| *Healthcoin* | ***A new tradable currency to finance breakthrough medicines. It is exchangeable for fiat currency. Used to compensate for patients transitioning from a private insurer into the public system, when the first had paid before for instance for a curative therapy. Aim: to incentivise private payers to invest in breakthrough therapies.*** | ***Payer/s (public), Insurers (private)*** | ***United States*** | ***(Hanna et al., 2018, Basu et al., 2016)*** |

| **ANNEX IV – Survey questions** |
| --- |

We have transcribed the questions we included in the survey into this annex. The questions included were:

- Is this ATMP financed or reimbursed in your health system?
- If not financed, please give reasons
- Is it financed under special arrangements (managed entry agreement (MEA), patient access scheme (PAS) or other)?
- What is the main purpose of the MEA (e.g. control expenditure, generate further evidence, share risk, other)?
- What type of MEA (confidential discount, volume or expenditure cap, free initial treatment, payment by results, other)?
- Is information on the MEA publicly available?
- Where can the information be found? (link)
- Is the MEA linked to the collection of further evidence?
- What is the nature of the data collected for the MEA and what database/s are used?
- Who is responsible for collecting this information (manufacturer, HTA agency, health ministry, other)?
- Who is responsible for analysing this information (manufacturer, HTA agency, health ministry, other)?
- Is the reassessment of the evidence, coverage or price planned? Please describe the conditions and timelines (e.g., yearly, once after 3 years maximum, etc.)?
- Optional: any further information?

| **ANNEX V – Medicine regulators, HTA agencies and competent authorities of included countries** |
| --- |

|  | **Medicines regulator** | **HTA agency** |
| --- | --- | --- |
| Armenia | Scientific Centre of Drug and Medical Technology Expertise (SCDMTE) | No use of HTA for pricing and/or reimbursement decisions (Vogler, 2022) |
| Australia | Therapeutic Goods Administration | Pharmaceutical Benefits Advisory Committee |
| Brazil | Brazilian Health Regulatory Agency (Anvisa) | National Committee for Technology Incorporation (CONITEC) |
| Canada | Health Products and Food Branch (HPFB) of Health Canada | Canada’s Drug and Health Technology Agency (CADTH) |
| Israel | Pharmaceutical Division, within the Israeli Ministry of Health | Israeli Center for Technology Assessment in Health Care (ICTAHC) |
| Türkiye | Turkish Medicines and Medical Devices Agency (TMMDA) (Atikeler et al., 2022) | Health Services General Directorate - Research, Development and Health Technology Evaluation Department within the Turkish Ministry of Health (Atikeler et al., 2022) |
| Austria | EMA | Austrian Institute for Health Technology Assessment (AIHTA) |
| Bulgaria | EMA | National Centre for Public Health and Analyses (NCPHA) |
| Czechia | EMA | the State Institute for Drug Control – SÚKL is its acronym in Czech |
| Denmark | EMA | Danish Medicines Council |
| France | EMA | Haute Autorité de santé, or HAS |
| Germany | EMA | Institute for Quality and Efficiency in Health Care (IQWiG) – usually commissioned by the GBA (Federal Joint Committee or Gemeinsamer Bundesausschuss) |
| Greece | EMA | HTA and reimbursement committee for medicinal products for  human use within the National Organization for Medicines (EOF) (BARBOSA et al., 2022) |
| Iceland | EMA | The Icelandic Medicine Pricing and Reimbursement Committee (IMPRC) (Elena Nicod, 2020a) |
| Malta | EMA | Health Technology Assessment (HTA) Unit within the Health System’s Directorate for Pharmaceutical Affairs (DPA) (Elena Nicod, 2020b) |
| Netherlands (Kingdom of the) | EMA | National Health Care Institute (ZIN) |
| Slovenia | EMA | Agency for Medicinal Products and Medical Devices (JAZMP) |
| Spain | EMA | Interministerial Committee for Pricing and Reimbursement |
| Sweden | EMA | Dental and Pharmaceutical Benefits Board (TLV) |

| **ANNEX VI – Glossary of therapies and indications** |
| --- |

| ***ATMP*** | ***Indication*** | ***Abbreviation used in our manuscript*** |
| --- | --- | --- |
| ATMP1 | Multiple myeloma | MM or indication 1 (I1) |
| ATMP2 | Anal fistulas in Crohn's disease patients | AFCDP or indication 2 (I2) |
| ATMP3 | Replacement of damaged corneal cells | RDCC or indication 3 (I3) |
| ATMP4 | Melanoma not removable by surgery | MNRBS or indication 4 (I4) |
| ATMP5 | B-cell acute lymphoblastic leukemia | BCALL or indication 5 (I5) |
| ATMP5 | Diffuse large B-cell lymphoma | DLBCL or indication 6 (I6) |
| ATMP6 | Metachromatic leukodystrophy | ML or indication 7 (I7) |
| ATMP7 | Hereditary retinal dystrophy | HRD or indication 8 (I8) |
| ATMP8 | Knee cartilage repair | KCR or indication 9 (I9) |
| ATMP9 | Severe combined immunodeficiency due to adenosine deaminase deficiency | SCIDTADD or indication 10 (I10) |
| ATMP10 | Mantle cell lymphoma | MCL or indication 11 (I11) |
| ATMP11 | Diffuse large B-cell lymphoma | DLBCL or indication 12 (I12) |
| ATMP11 | Primary mediastinal lymphoma | PML or indication 13 (I13) |
| ATMP12 | Spinal muscular atrophy | SMA or indication 14 (I14) |
| ATMP13 | Beta thalassaemia | BT or indication 15 (I15) |

**References for supplementary material**

ANDERSON, M., DRUMMOND, M., TAYLOR, D., MCGUIRE, A., CARTER, P. & MOSSIALOS, E. 2022. Promoting innovation while controlling cost: The UK's approach to health technology assessment. *Health Policy,* 126**,** 224-233.

ANDERSSON, E., SVENSSON, J., PERSSON, U. & LINDGREN, P. 2020. Risk sharing in managed entry agreements—A review of the Swedish experience. *Health Policy,* 124**,** 404-410.

ATIKELER, E. K., FASSEEH, A. N., MANTEL-TEEUWISSE, A. K., ÇALıŞKAN, Z., ÖNER, Z. G., KıZıLAY, H., KALO, Z. & GOETTSCH, W. 2022. Health technology assessment in Türkiye: Current status and perspectives on future implementation. *Health Policy and Technology***,** 100701.

BABAR, Z.-U.-D. & ATIF, M. 2014. Differential pricing of pharmaceuticals: a bibliometric review of the literature. *Journal of Pharmaceutical Health Services Research,* 5**,** 149-156.

BARBOSA, M. M., ZAMPIROLLI, C. D., ZUPPO, I. F., NASCIMENTO, R. M., KRITIKOU, P., ACURCIO, F. A. & ÁLVARES-TEODORO, J. 2022. Regulatory intelligence of health technologies in Greece. *Revista Brasileira de Farmácia Hospitalar e Serviços de Saúde,* 13**,** 789-789.

BASU, A., SUBEDI, P. & KAMAL-BAHL, S. 2016. Financing a cure for diabetes in a multipayer environment. *Value in Health,* 19**,** 861-868.

BRÜGGER, U., RUCKSTUHL, A., HORISBERGER, B. & GRATWOHL, A. 2014. Development of coverage with evidence development for medical technologies in Switzerland from 1996 to 2012. *International Journal of Technology Assessment in Health Care,* 30**,** 253-259.

CARL, D. L. & VOKINGER, K. N. 2021. Patients’ access to drugs with rebates in Switzerland–Empirical analysis and policy implications for drug pricing in Europe. *The Lancet Regional Health-Europe,* 3**,** 100050.

CARR, D. R. & BRADSHAW, S. E. 2016. Gene therapies: the challenge of super-high-cost treatments and how to pay for them. *Regenerative medicine,* 11**,** 381-393.

CLOPES, A., GASOL, M., CAJAL, R., SEGÚ, L., CRESPO, R., MORA, R., SIMON, S., CORDERO, L. A., CALLE, C. & GILABERT, A. 2017. Financial consequences of a payment-by-results scheme in Catalonia: gefitinib in advanced EGFR-mutation positive non-small-cell lung cancer. *Journal of medical economics,* 20**,** 1-7.

COYLE, D., DURAND-ZALESKI, I., FARRINGTON, J., GARRISON, L., GRAF VON DER SCHULENBURG, J.-M., GREINER, W., LONGWORTH, L., MEUNIER, A., MOUTIÉ, A.-S. & PALMER, S. 2020. HTA methodology and value frameworks for evaluation and policy making for cell and gene therapies. *The European Journal of Health Economics,* 21**,** 1421-1437.

DABBOUS, M., CHACHOUA, L., CABAN, A. & TOUMI, M. 2020. Managed entry agreements: policy analysis from the European perspective. *Value in Health,* 23**,** 425-433.

DIEGO, B. 2015. *Prices of new hepatitis C combinations lower than monotherapy in Spain in six months* [Online]. APM Health Europe. Available: <https://www.apmhealtheurope.com/libre_story.php?numero=41696> [Accessed].

ELENA NICOD, A. W., KAREN FACEY 2020a. IMPACT-HTA: Country vignette of appraisal/reimbursement processes for rare disease medicines in Iceland.

ELENA NICOD, A. W., KAREN FACEY 2020b. IMPACT-HTA: Country vignette of appraisal/reimbursement processes for rare disease medicines in Malta.

FULLER, R. L. & GOLDFIELD, N. 2016. Paying for On-Patent Pharmaceuticals: limit prices and the emerging role of a pay for outcomes approach. *The Journal of Ambulatory Care Management,* 39**,** 143.

GARRISON JR, L. P., TOWSE, A., BRIGGS, A., DE POUVOURVILLE, G., GRUEGER, J., MOHR, P. E., SEVERENS, J. H., SIVIERO, P. & SLEEPER, M. 2013. Performance-based risk-sharing arrangements—good practices for design, implementation, and evaluation: report of the ISPOR good practices for performance-based risk-sharing arrangements task force. *Value in Health,* 16**,** 703-719.

GAVIOUS, A., GREENBERG, D., HAMMERMAN, A. & SEGEV, E. 2014. Impact of a financial risk-sharing scheme on budget-impact estimations: a game-theoretic approach. *The European Journal of Health Economics,* 15**,** 553-561.

HAMPSON, G., TOWSE, A., PEARSON, S. D., DREITLEIN, W. B. & HENSHALL, C. 2018. Gene therapy: evidence, value and affordability in the US health care system. *Journal of comparative effectiveness research,* 7**,** 15-28.

HANNA, E., TOUMI, M., DUSSART, C., BORISSOV, B., DABBOUS, O., BADORA, K. & AUQUIER, P. 2018. Funding breakthrough therapies: a systematic review and recommendation. *Health Policy,* 122**,** 217-229.

HUSSEY, P. S., RIDGELY, M. S. & ROSENTHAL, M. B. 2011. The PROMETHEUS bundled payment experiment: slow start shows problems in implementing new payment models. *Health Affairs,* 30**,** 2116-2124.

JØRGENSEN, J., HANNA, E. & KEFALAS, P. 2020. Outcomes-based reimbursement for gene therapies in practice: the experience of recently launched CAR-T cell therapies in major European countries. *Journal of market access & health policy,* 8**,** 1715536.

JØRGENSEN, J. & KEFALAS, P. 2017. Annuity payments can increase patient access to innovative cell and gene therapies under England’s net budget impact test. *Journal of market access & health policy,* 5**,** 1355203.

JØRGENSEN, J. & KEFALAS, P. 2021. The use of innovative payment mechanisms for gene therapies in Europe and the USA. *Regenerative medicine,* 16**,** 405-422.

KAMUSHEVA, M. S., TURCU-STIOLICA, A., GIERCZYŃSKI, J., SUBTIRELU, M.-S., CZECH, M. & PETROVA, G. I. 2021. Do advanced therapies have a future in the low-and middle-income countries-the case with Bulgaria, Romania and Poland. *Frontiers in Public Health***,** 1215.

KLEINKE, J. & MCGEE, N. 2015. Breaking the bank: three financing models for addressing the drug innovation cost crisis. *American health & drug benefits,* 8**,** 118.

LEE, B., BAE, E.-Y., BAE, S., CHOI, H.-J., SON, K.-B., LEE, Y.-S., JANG, S. & LEE, T.-J. 2021. How can we improve patients’ access to new drugs under uncertainties?: South Korea’s experience with risk sharing arrangements. *BMC health services research,* 21**,** 1-12.

MASINI, C., GALLEGATI, D., GENTILI, N., MASSA, I., CIUCCI, R. & ALTINI, M. 2021. The Challenge of Sustainability of High-Cost Oncological Drugs: A Budgeting Model in an Italian Cancer Center. *International Journal of Environmental Research and Public Health,* 18**,** 13413.

MEGHANI, A. & BASU, S. 2015. A review of innovative international financing mechanisms to address noncommunicable diseases. *Health Affairs,* 34**,** 1546-1553.

MESSORI, A. 2016a. Application of the Price–Volume approach in cases of innovative drugs where value-based pricing is inadequate: description of real experiences in Italy. *Clinical drug investigation,* 36**,** 599-603.

MESSORI, A. 2016b. Evolocumab and alirocumab: exploring original procurement models to manage the reimbursement of these innovative treatments. *Int J Clin Pharmacol Ther,* 54**,** 771-4.

MICALLEF, J. & BLIN, O. 2020. Orphan drug designation in Europe: A booster for the research and development of drugs in rare diseases. *Therapies,* 75**,** 133-139.

MILLS, M. & KANAVOS, P. 2020. Do pharmaceutical budgets deliver financial sustainability in healthcare? Evidence from Europe. *Health Policy,* 124**,** 239-251.

MOON, S. & ERICKSON, E. 2019. Universal medicine access through lump-sum remuneration—Australia’s approach to hepatitis C. *New England Journal of Medicine,* 380**,** 607-610.

PERSSON, U., SVENSSON, J. & PETTERSSON, B. 2012. A new reimbursement system for innovative pharmaceuticals combining value-based and free market pricing. *Applied health economics and health policy,* 10**,** 217-225.

PRECKLER, V. & ESPÍN, J. 2022. The role of indication-based pricing in future pricing and reimbursement policies: a systematic review. *Value in Health*.

TAKAYAMA, A. & NARUKAWA, M. 2016. Pharmaceutical pricing and reimbursement in Japan: for faster, more complete access to new drugs. *Therapeutic Innovation & Regulatory Science,* 50**,** 361-367.

TRUSHEIM, M. R., CASSIDY, W. M. & BACH, P. B. 2018. Alternative State-Level Financing for Hepatitis C Treatment—The “Netflix Model”. *JAMA,* 320**,** 1977-1978.

VOGLER, S. 2022. Payer policies to support innovation and access to medicines in the WHO European Region.

VOGLER, S., ZIMMERMANN, N., HABL, C., PIESSNEGGER, J. & BUCSICS, A. 2012. Discounts and rebates granted to public payers for medicines in European countries. *Southern med review,* 5**,** 38.

VREMAN, R. A., BROEKHOFF, T. F., LEUFKENS, H. G., MANTEL-TEEUWISSE, A. K. & GOETTSCH, W. G. 2020. Application of Managed Entry Agreements for Innovative Therapies in Different Settings and Combinations: A Feasibility Analysis. *Int J Environ Res Public Health,* 17.

WENZL, M. & CHAPMAN, S. 2019. Performance-based managed entry agreements for new medicines in OECD countries and EU member states: How they work and possible improvements going forward. *OECD Health Working Papers.*

WHO 2016. Challenges and opportunities in improving access to medicines through efficient public procurement in the WHO European Region.

WHO 2020. WHO guideline on country pharmaceutical pricing policies. Second edition ed.: World Health Organization.

ZETTLER, P. J. & EC, F. B. 2017. The challenge of paying for cost-effective cures. *The American Journal of Managed Care,* 23**,** 62-64.

1. All references to Kosovo in this document should be understood to be in the context of the United Nations Security Council resolution 1244 (1999). [↑](#footnote-ref-1)
2. All references to Kosovo in this document should be understood to be in the context of the United Nations Security Council resolution 1244 (1999). [↑](#footnote-ref-2)
